# Adherence to the Mediterranean Diet in medical students

**DOI:** 10.1017/S1368980023000964

**Published:** 2023-09

**Authors:** Sonia González-Sosa, Jose Juan Ruiz-Hernández, Alicia Puente-Fernández, José María Robaina-Bordón, Alicia Conde-Martel

**Affiliations:** 1 Department of Internal Medicine, Hospital Universitario de Gran Canaria Doctor Negrín, Las Palmas de Gran Canaria, Barranco La Ballena s/n 35012 Las Palmas, GC, Spain; 2 Faculty of Health Sciences, Universidad de Las Palmas de Gran Canaria, Las Palmas de Gran Canaria, Paseo Blas Cabrera Felipe, s/n. 35016 Las Palmas, GC, Spain

**Keywords:** Mediterranean Diet, Nutrition, Food habits, Lifestyle behaviours, University students

## Abstract

**Objective:**

The protective effect of the Mediterranean Diet (MeDi) is undisputed. However, adherence to MeDi has decreased in recent years, particularly in young people. The aim of this study was to evaluate adherence to MeDi in medical students and to assess the influence of knowledge acquisition as well as other factors on dietary compliance.

**Design:**

A cross-sectional study was conducted on medical students. The data were obtained through anonymous surveys that collected demographic characteristics, medical history, alcohol and tobacco consumption, physical activity and adherence to MeDi – using 14-point Mediterranean Diet Adherence Score (MEDAS) –. Adherence to MeDi and related factors were evaluated by univariate and multivariable analysis.

**Participants:**

Medical students from the first to the sixth year of the 2018–2019 academic year.

**Setting:**

The study was conducted at the university of Las Palmas de Gran Canaria.

**Results:**

Of 589 respondents (73 % women) mean aged 22 years (range 18–39), 58·9 % showed good adherence to MeDi. Adherence was significantly associated with age (*P* = 0·017) but not with sex or the presence of comorbidities. Independently, adherence to MeDi was higher in last academic courses (OR = 2·1; 95 % CI = 1·3, 3·2; *P* = 0·001), in those who consumed alcohol more frequently (OR = 1·5; 95 % CI = 1·0, 2·1; *P* = 0·039) and in those who practiced more exercise (OR = 1·5; 95 % CI = 1·2, 1·9; *P* < 0·001).

**Conclusions:**

Half of all medical students did not have a good adherence to MeDi. Adherence was higher at older age in higher academic years and related to greater physical activity. It would be convenient to quantify dietary knowledge as well as implement nutritional educational programmes, favouring a healthy lifestyle.

## Graphical abstract

**Figure:**
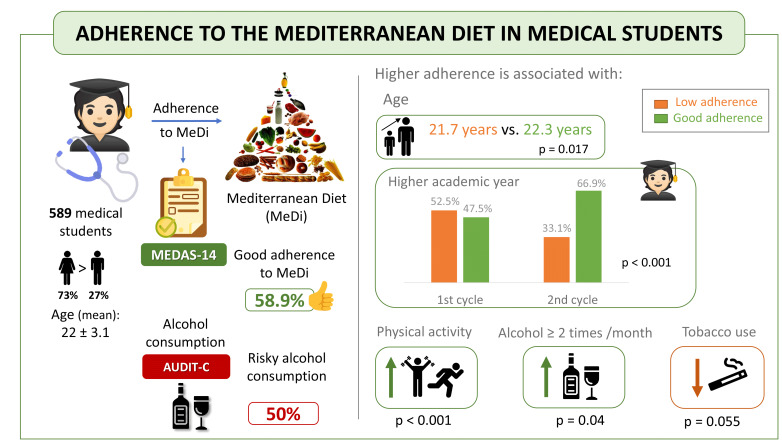

The concept of Mediterranean Diet (MeDi) was introduced in the late 1950s, describing the dietary habits found throughout the Mediterranean coast^(1)^. Traditional MeDi is characterised by a high consumption of vegetables, fruits, legumes, nuts, unrefined cereals, fish and olive oil and a low consumption of dairy products and red meat^(2,3)^. The Mediterranean dietary pattern has been consistently shown to provide a grade of protection against CVD and main non-infectious chronic diseases, such as cancer or diabetes^(2–7)^. A wide range of studies support MeDi as an effective preventive tool to reduce morbidity and mortality in the general population^(2,8,9)^.

MeDi compliance has declined dramatically in recent years^(5,8)^. Particularly, young adults are strongly influenced by socio-cultural changes and tend to develop unhealthy eating habits and decrease their MeDi consumption^(10–13)^. There are multiple factors that affect the quality of diet: demographic characteristics, unhealthy behaviours – such as sedentary lifestyle – and family and social influence^(14)^. In fact, a possible association has been established between the social context – eating more frequently outside home and accompanied – and changes in dietary habits^(15)^. In addition, alcohol consumption is an important aspect in undergraduate students, a population prone to risky alcohol consumption^(16,17)^.

Although measuring the level of adherence to a Mediterranean dietary pattern is not easy, it is of great importance for dietary advice in routine clinical practice^(18)^. For this purpose, diet quality indices have been proposed to assess the degree of adherence to MeDi and its health benefits^(19,20)^.

On the other hand, it has been shown that increased nutritional knowledge is associated with improved dietary habits^(5,21,22)^. Likewise, it is assumed that greater knowledge and better habits influence a better transmission of nutritional advice^(23,24)^. The latter is increasingly required in health professionals due to the previously described. Adherence to MeDi by medical students has been little studied^(21,25)^. Their knowledge is of interest because, in addition to assessing the MeDi compliance of university students, they are future health professionals.

The purpose of this study is to evaluate the use of MeDi by medical undergraduate students and to analyse the influence of knowledge acquired over the years of study as well as other factors on dietary compliance.

## Material and methods

### Study design

This is a cross-sectional observational study to assess adherence to MeDi in medical students.

### Population, study scope and recruitment

The study population included students enrolled in the medical degree of the Faculty of Health Sciences of the University of Las Palmas de Gran Canaria. It involved students from first to sixth year of the 2018–2019 academic year. Likewise, the candidates to medical resident interns of the province of Las Palmas of the same year. The only inclusion criteria was to belong to one of these courses at the time of the study. The only exclusion criteria was a refusal to participate. A survey was carried out and sent by e-mail and handed out on paper to all students. All students who responded to the survey (61·85 %) were included.

### Variables of interest and data collection

A survey was carried out (see Appendix 1), collecting data on age, sex, weight and height as self-referenced. BMI was estimated and categorised according to WHO criteria as: underweight (IMC < 18·5 kg/m^2^), normal weight (IMC ≥ 18·5 years < 25 kg/m^2^), overweight (IMC ≥ 25 years < 30 kg/m^2^) and obese (IMC ≥ 30 kg/m^2^)^(26)^. In addition, personal medical history (hypertension, diabetes, dyslipidaemia…) and lifestyle-related variables such as physical activity and alcohol-tobacco consumption are detailed. In relation to physical activity, the fundamental components of the training load^(27)^ are included: (1) the frequency with which physical activity is performed – none, 1–2 sessions per week or greater than or equal to 3 sessions; (2) the volume of time per session – less than 30 min, 30 min to one hour or more than 1 hour and (3) the subjective intensity of the exercise performed – light, moderate, intense or maximum. Alcohol consumption was also collected, including questions from the AUDIT-C questionnaire^(28–30)^, a short version of the AUDIT test consisting of the first three items of the AUDIT, including frequency and amount of alcohol consumption and frequency of binge drinking. The AUDIT-C cut-off point of ≥ 4 in men and ≥ 3 in women was used to identify hazardous alcohol consumption^(30)^. The 14-point Mediterranean Diet Adherence Score (MEDAS-14) questionnaire, widely validated for the Spanish population^(20)^ and in its English version^(31)^ and simple to complete,^(2,20)^ was used to record adherence to MeDi. The MEDAS-14 includes fourteen dichotomous response questions (yes/no) and the total adherence score ranges from a minimum of zero points to a maximum of fourteen and a score of nine points or more is considered good diet adherence^(20)^.

The questionnaires were completed through anonymous and voluntary surveys carried out in paper or electronic format. It was necessary to contact them in person or through institutional mail. If no initial response was obtained, up to three contacts were made.

### Ethical considerations

This study was authorised by the Ethics and Clinical Research Committee of the University Hospital of Gran Canaria Doctor Negrín. Likewise, the approval of the Faculty of Health Sciences of the University of Las Palmas de Gran Canaria was also granted.

### Statistical analysis

The data were analysed with the statistical package IBM SPSS software (IBM Corp. Released 2011. IBM SPSS Statistics for Windows, Version 26.0., IBM Corp.). Categorical variables are expressed as percentages and quantitative variables as mean and sd or median and interquartile range, depending on whether or not the distribution was normal. Normality of the quantitative variables was assessed using the Kolmogorov–Smirnov test.

To evaluate the relationship between qualitative variables, the Chi-square test or Fisher’s exact test was used, and for the association between quantitative variables and MeDi compliance, the Student’s *t* test or the Mann–Whitney *U* test was used, depending on whether or not the variables followed a normal distribution. To identify the variables independently associated with MeDi, a multivariable analysis was performed using logistic regression in which the variables that were significantly related in the univariate analysis were included. These variables were age, gender, academic year, physical activity, alcohol and tobacco consumption. Differences with a *P* value < 0·05 were considered significant.

## Results

Of 589 medical students included in the study, 430 (73 %) were female and 159 (27 %) were male with a mean age of 22·1 years (sd: 3·1), range: 18 to 39, median 22 (interquartile range: 22–24).

From the total, 242 (41·1 %) were in the first to third year of their degree course and 347 (58·9 %) were in the second cycle, including resident opponents. The distribution according to academic year is detailed in Table 1.

**Table 1:** Distribution of students according to academic course

Academic course	Frequency		Age		Women	
	Abs freq	%	Median	IQR	*n*	%
1st year	79	13·4	18	18–18	60	75·9
2nd year	65	11·0	19	19–20	51	78·5
3rd year	98	16·6	20	20–21	67	68·4
4th year	58	9·8	21	21–23	46	79·3
5th year	85	14·4	22	22–23	62	72·9
6th year	116	19·7	23	23–24	78	67·2
MIR	88	14·9	24	24–25	66	75·0
Total	589	100	22	20–24	430	73·0

Abs freq, absolute frequency; IQR, interquartilic range.

According to BMI, 70·6 % (416) of the subjects included in the study were normal weight, 9 % (53) were underweight, 15·1 % (89) were overweight and 5·3 % (31) were obese, i.e. 20·4 % (120) were overweight or obese.

In relation to cardiovascular risk factors, 8 (1·4 %) reported hypertension, 3 (0·5 %) diabetes and 3 (0·5 %) dyslipidemia.

Regarding smoking habit, 559 (94·9 %) respondents reported not smoking, 9 (1·5 %) were smokers and 21 (3·6 %) defined themselves as ex-smokers (≥ 1 year without smoking).

In relation to alcohol consumption, the mean AUDIT-C score was 2·74 (± 2·01), being higher in males (3·4 ± 2·3) compared to females (2·5 ± 1·8) (*P* < 0·001). Seventy-nine percent of the students consumed alcohol at least once a month, and one-third (35·3 %) between 2 and 4 times a month, with 5 % consuming alcohol four times a week or more. The amount consumed per occasion was at least 3–4 drinks in one-third of the cases (32·4 %), with 8·5 % consuming more than five drinks. Half of the students (49·9 %) had risky alcohol consumption, with no differences according to sex (50·5 % in females and 48·4 % in males, *P* = 0·66), or age (22·0 *v*. 22·1 years; *P* = 0·84). However, a significant association was observed between risky alcohol consumption and tobacco consumption (80 % *v*. 48·3 %; *P* = 0·001; OR: 4·3, 95 % CI: 1·7, –10·6), and belonging to the highest medical school grades (5th or 6th grade *v*. 1st to 4th grades; 57·2 *v*. 45·3; *P* = 0·007; OR: 1·6, 95 % CI: 1·1, –2·3).

When it comes to physical activity, 71 % exercised at least once a week compared with 29 % who did not exercise regularly. The amount of time spent was less than 30 min per session in 10·7 %, from 30 min to 1 hour in 42·1 % and more than 1 hour in 23·8 %. Finally, exercise intensity was mild in 8·8 %, moderate in 39·4 % and intense maximum in 29·4 %.

The majority of the respondents (76·1 %) did not usually cook at home. There were a tendency to cook at home in second-cycle students compared to first-cycle students (28·7 % *v*. 20·2 %; *P* = 0·07).

Adherence to MeDi was acceptable in 58·9 % students. Table 2 shows the individualised analysis of the MEDAS-14 questions, globally and by sex. The mean MEDAS-14 score obtained was 8·9 (±1·9) points, with no differences according to sex (8·9 (±1·9) in women and 8·72 (± 2·0) in men (*P* = 0·46)). Fish or seafood consumption was significantly higher in men (42·1 % *v*. 25·1 %; *P* < 0·001). Consumption of white meat was higher in women (85·6 % *v*. 77·4 %; *P* = 0·017) and women consumed significantly less red meat (76·7 % women consumed less than once a day *v*. 68·6 % men; *P* = 0·043). Women also tended almost significantly to a higher consumption of vegetables (62·3 % *v*. 53·5 %; *P* = 0·051). There were no significant differences in the rest of the MEDAS-14 questions according to sex.

**Table 2:** Answer to each MEDAS-14 individual question and differences between women and men

Questions of MEDAS-14	Agreement with the recomendation						
	Total (*n* 589)		Women (*n* 430)		Men (*n* 159)		*P*
	*n*	%	*n*	%	*n*	%	
1. Use of olive oil for cooking:	569	96·6	416	96·7	153	96·2	0·758
2. Consumption of ≥ 2 total tablespoons of olive oil per day:	451	76·6	326	75·8	125	78·6	0·476
3. Consumption of ≥ 2 servings of vegetables per day:	353	59·9	268	62·3	85	53·5	0·051
4. Consume ≥ 3 pieces of fruit per day:	242	41·1	175	40·7	67	42·1	0·752
5. Consumption of < 1 serving of red meat, hamburgers, sausages per day:*	439	74·5	330	76·7	109	68·6	0·043
6. Consumption of < 1 serving of butter, margarine, or cream per day:	470	79·8	350	81·4	120	75·5	0·112
7. Consumption of < 1 carbonated and/or sweetened beverage per day:	433	73·5	320	74·4	113	71·1	0·413
8. Consumption of ≥ 3 glasses of wine per week:	15	2·5	9	2·1	6	3·8	0·250
9. Consumption of ≥ 3 servings of legumes per week:	307	52·1	219	50·9	88	55·3	0·341
10. Consumption of ≥ 3 servings of fish or seafood per week:*	175	29·7	108	25·1	67	42·1	<0·001
11. Consumption of ≤ 3 industrial pastries per week:	373	63·3	278	64·7	95	59·7	0·273
12. Consumption of ≥ 1 nuts servings per week:	377	64·0	278	64·7	99	62·3	0·592
13. Preferable consumption of chicken, turkey, or rabbit before beef, pork, hamburgers, or sausages:*	491	83·4	368	85·6	123	77·4	0·017
14. Consumption of ≥ 2 times per week of meals dressed with *sofrito*:	500	84·9	363	84·4	137	86·2	0·600

* *P* < 0·05.

### Relationship between the different variables and adherence to Mediterranean Diet

As shown in Table 3, adherence to MeDi was not related to sex; nevertheless, greater adherence to MeDi was observed at older age (*P* = 0·017).

**Table 3:** Relation between Mediterranean adherence and demographic characteristics, academic course, tobacco use and culinary habits

	Low adherence (*n* 242)		Good adherence (*n* 347)		*P* value	OR	95 % CI
	*n*	%	*n*	%			
Demographic data							
Females	176	72·7	254	73·2	0·899	0·97	0·66, 1·41
Males	66	27·3	93	26·8			
Age							
Mean	21·7		22·32		0·017	—	
sd	3·29		2·94				
Academic course							
1st year	49	62·0	30	38·0	<0·001	—	
2nd year	22	33·8	43	62·2			
3rd year	56	57·1	42	42·9			
4th year	25	43·1	33	56·9			
5th year	27	31·8	58	68·2			
6th year	43	37·1	73	62·9			
MIR	20	22·7	68	77·3			
1st cycle (1st–3rd year)	127	52·5	115	47·5	<0·001	2·23	1·59, 3·12
2nd cycle (≥ 4rd year)	115	33·1	232	66·9			
BMI							
BMI ≥ 25	56	23·1	64	18·4	0·164	0·75	0·5, 1·12
Tobacco use							
Smoker-former smoker	17	7·0	13	3·8	0·063	0·52	0·25, 1·08
Culinary habits							
Cooks at home	53	21·9	88	25·4	0·333	1·21	0·82, 1·78

sd, standard deviation.

**Fig. 1:**
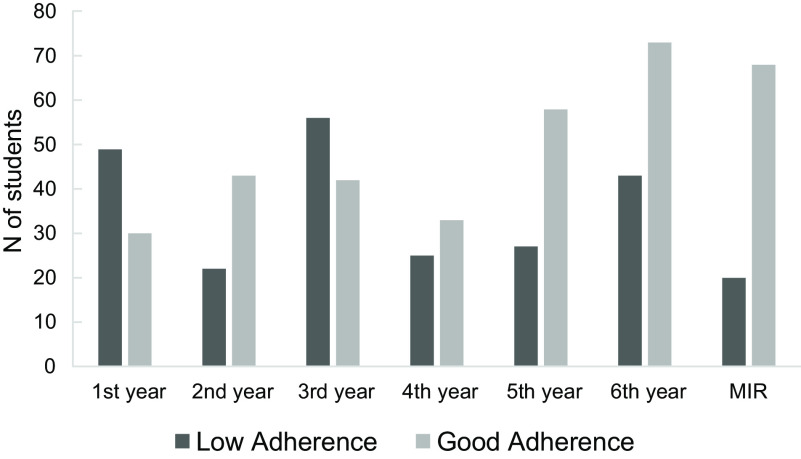
Differences in Mediterranean Diet adherence by academic years

In the analysis by academic year (Fig. 1), MeDi consumption was higher in students in the second cycle (fourth to sixth year of studies and medical opponents) compared to students in the first three years (OR = 2·3; 95 % CI: 1·6, 3·1; *P* < 0·001).

No higher adherence to MeDi was observed in those who cooked at home compared to those who did not. Nor was it observed in normal-weight patients with respect to overweight or obese patients.

In relation to tobacco consumption, there was no statistically significant but close relationship (*P* = 0·06) in favour of a better dietary habit in non-smokers.

The frequency of alcohol consumption was associated with adherence to MeDi (*P* = 0·008): students who consumed alcohol two or more times per month showed a significantly higher adherence to MeDi (*P* = 0·002) (Table 4). However, MeDi compliance was not associated with the amount consumed per occasion (*P* = 0·350).

**Table 4:** Relation between Mediterranean adherence and physical activity and alcohol consumption

		Total		Low adherence (*n* 242)		Good adherence (*n* 347)		*P* value
		*n*	%	*n*	%	*n*	%	
Physical activity								
Frequency	None	171	29·0	97	40·1	74	21·3	< 0·001
	1–2 session/week	208	35·3	72	29·8	136	39·2	
	≥ 3 session/week	210	35·7	73	30·2	137	39·5	
Volume	None	138	23·4	75	31·0	63	18·2	0·001
	< 30 min/session	63	10·7	27	11·2	36	10·4	
	30min–1 h	248	42·1	97	40·1	151	43·5	
	> 1 h/session	140	23·8	43	17·8	97	28·0	
Intensity	None	132	22·4	71	29·3	61	17·6	0·003
	Mild	52	8·8	19	7·9	33	9·5	
	Moderate	232	39·4	97	40·1	135	38·9	
	Intense	163	27·7	53	21·9	110	31·7	
	Maximum	10	1·7	2	0·8	8	2·3	
Alcohol consumption								
AUDIT-C	Mean	2·74		2·64		2·82		0·29
	sd	2·01		1·94		2·06		
AUDIT-C	Low-risk drinking	295	50·1	126	52,1	169	48,7	0·42
	Hazardous drinking	294	49·9	116	47·9	178	51,3	
Frequency of consumption	Never	124	21·1	53	21·9	71	20·5	0·008
	≤ 1/month	229	38·9	110	45·5	119	34·3	
	2–4/month	208	35·3	74	30·6	134	38·6	
	2–3/week	27	4·6	5	2·1	22	6·3	
	≥ 4/week	1	0·2	0	0·0	1	0·3	
Drinks per occasion	0 drinks	217	36·8	86	35·5	131	37·8	0·350
	1–2 drinks	181	30·7	67	27·7	114	32·9	
	3–4 drinks	141	23·9	66	27·3	75	21·6	
	5–6 drinks	40	6·8	20	8·3	20	5·8	
	7–9 drinks	8	1·4	2	0·8	6	1·7	
	≥ 10 drinks	2	0·3	1	0·4	1	0·3	
Frequency of ≥ 6 consumptions	Never	376	63·8	161	66·5	215	62·0	0·215
	< 1/month	171	29	70	28·9	101	29·1	
	Monthly	36	6·1	9	3·7	27	7·8	
	Weekly	6	1	2	0·8	4	1·2	

AUDIT-C, Alcohol Use Disorder Identification Test–Consumption.

AUDIT-C cut-off = low-risk drinking (score < 3 in women and < 4 in men) and hazardous drinking (score ≥ 3 in women and ≥ 4 in men).

**Fig. 2:**
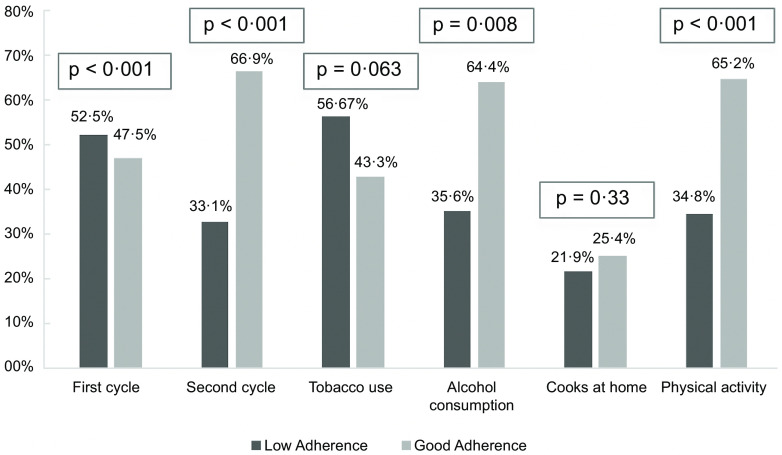
Most relevant variables associated to Mediterranean Diet

Adherence to MeDi was highly significantly associated (*P* < 0·001) with the performance of physical activity (Table 4), both with frequency (*P* < 0·001), intensity (*P* = 0·003) and volume of exercise performed (*P* = 0·001). Figure 2 represents a summary of the results described above in a more visual form.

### Multivariable analysis

The significant association between greater adherence to MeDi in second-cycle students compared to first-cycle students was maintained (OR = 2·1; 95 % CI = 1·3, 3·2; *P* = 0·001). Similarly, MeDi consumption was higher in those who consumed alcohol two or more times a month (OR = 1·5; 95 % CI = 1·0, 2·1; *P* = 0·039) and those who were physically active (OR = 1·5; 95 % CI = 1·2, 1·9; *P* < 0·001). The remaining variables were not independently associated with MeDi compliance although tobacco use showed a trend toward statistical significance in its negative association with MeDi consumption (OR = 0·5; 95 % CI: 0·2, 1·01; *P* = 0·055) (Table 5).

**Table 5:** Multivariable analysis to evaluate the factors independently associated with adherence to the Mediterranean Diet

	*β* coefficient	*P* value	OR	IC 95 %
Age	−0·003	0·94	0·99	0·93–1·07
Sex	−0·21	0·31	0·82	0·55–1·2
Academic course (≥ 4th year)	0·72	0·001	2·05	1·32–3·18
Physical activity	0·39	< 0·001	1·48	1·19–1·85
Tobacco consumption	−0·78	0·055	0·46	0·21–1·02
Alcohol consumption	0·39	0·04	1·47	1·02–2·13
Constant	−0·25	0·74	—	

## Discussion

Adherence to MeDi was acceptable in 59 % of undergraduate medical students. This degree of adherence, despite not being optimal, is notably better than that obtained in other studies in Spanish undergraduates in general, which show good adherence in only 36 %^(32)^ and 34 %^(33)^, using the same MEDAS-14 questionnaire. Other studies using different questionnaires have also observed a low percentage of university students with good compliance^(11)^.

This difference could be justified by the evidence that future physicians, compared to the overall university population, probably show greater concern for their health. Indeed, it is worth noting that MeDi compliance was significantly higher in students with higher grades. In this case, since these are medical students, it could be considered that nutrition knowledge may be increased. This could support that higher MeDi knowledge is related to higher MeDi adherence. In fact, there are studies that support that teaching in nutrition-food subjects implies greater adherence to MeDi^(22)^. An Italian study that evaluated nutritional knowledge and its association with adherence to MeDi showed a significant association between both: the greater the nutritional knowledge, the greater the adherence to MeDi^(34)^. Furthermore, a study that compared differences in terms of adherence to MeDi in health science students during their first academic year with respect to the second found greater adherence at the end of the second academic year, which could be justified by greater knowledge about dietary habits^(35)^. Nevertheless, another study that analysed adherence to MeDi in medical students in Italy did not observe that being in the first or last courses influenced the results^(21)^.

Likewise, in our study, adherence to MeDi increases significantly with age. This association has also been described recently in children and adolescents in Italy^(36)^, although contrary results, lower adherence at older age, are described in most studies included in a meta-analysis that also assessed adherence to MeDi in children and adolescents^(37)^.

It is worth emphasising that lifestyle habits are developed from childhood and become entrenched in adolescence. Diet of young people and especially of university students poses an important challenge, as it may involve major changes in their lifestyle. In fact, another systematic review confirmed that the diet of adolescents tends to be characterised by an unsatisfactory dietary intake^(38)^. The importance that nutritional education can have on medical students should be emphasised, given that physicians have a very important role and opportunity to advise their patients on diet.

In the individualised analysis of the MEDAS-14 questions, a low overall consumption of fish was observed, a fact previously noted in university students^(32,33)^, but it was significantly higher in men, contrary to what was described by Cobo-Cuenca^(33)^. It was also observed that women consume more white meat compared to men who consume significantly more red meat, hamburgers or sausages. A higher consumption of red meat has been previously described in men, as well as a higher consumption of vegetables in women^(39)^, an aspect that in this study was also found to be close to statistical significance. It has been described in the literature that in Western societies women tend to show better dietary habits and give more importance to body weight than men^(40)^. These aspects contributing to the better adherence to MeDi observed in several studies in young women^(36,41)^, as well as in medical students^(21)^. However, other studies show no differences according to sex^(37)^, as in our results. Similar findings, that gender does not influence adherence to MeDi have been observed in studies in university students in Spain^(32)^ and Lebanon^(42)^.

It is worth mentioning that the percentage of overweight obesity in our sample is 20·9 %, similar to other studies^(33)^. Even though this series did not find a relationship between overweight obesity and adherence to MeDi, another study in Italian adolescents found that good adherence to MeDi significantly reduced the likelihood of overweight obesity^(43)^. Other studies have reported weight gain in patients with increased adherence to MeDi mainly at the expense of lean mass^(33)^.

On the other hand, cooking at home was not related to adherence to MeDi. Some studies^(10,25)^ report that students who live away from home develop worse habits than those who live at home, associating this with a decrease in the intake of home-cooked meals. In a similar vein, a study showed that eating away from home was associated with a lower trend to consume vegetables, fruits and legumes and a higher predisposition to consume processed meat, salty snacks and carbonated beverages^(15)^.

About smoking, a trend toward greater adherence to MeDi was observed in non-smokers, probably in relation to a healthier lifestyle. Some authors describe the same association between a lack of adherence to a healthy diet and tobacco consumption^(44,45)^.

In relation to alcohol consumption, approximately one over three students (35·3 %) consume alcohol 2 to 4 times a month and 5 % 2–3 times a week. In this study, a greater adherence to MeDi was found in students with a higher frequency of alcohol consumption. This is somewhat expected since MeDi includes regular moderate ethanol consumption, mainly in the form of wine. A Spanish study of health science students found a strong association between adherence to MeDi and those who consumed alcohol such as wine or beer compared to both abstainers and consumers of other distilled beverages^(46)^. Regarding the benefit of moderate alcohol consumption, specifically wine, several authors report that despite discretely increasing the risk of some types of cancer, it is consistently associated with a reduction in cardiovascular risk and, therefore, has an overall protective effect on total mortality^(47)^. Nevertheless, other studies suggest that the protective associations between alcohol consumption and mortality may be attributable in part to inappropriate selection of the reference group and weak adjustment for confounding factors^(48)^. Therefore, there is controversy as to whether or not moderate alcohol consumption should be recommended as part of a balanced and healthy diet.

However, MeDi compliance was not associated with the beverages consumed per occasion. It is important to highlight a pattern of consumption characterised by drinking large amounts in short periods of time, or binge drinking, which is frequently observed in young people. This alcohol consumption deviates from MeDi drinking pattern and can have significant negative repercussions on health. In our study, an 8·5 % of the respondents were at risk for alcohol consumption. Two recent Spanish studies, one of them involving first-year university students^(49)^ and the other one all-year science students^(32)^, concluded that 16·9 % and 26·2 %, respectively, had a risky alcohol consumption. These percentages are higher than in our sample of medical students and correlate with that described in other studies on university students. In deed, in a systematic review assessing alcohol consumption in Irish and UK university students,^(16)^ over 20 % exceeded sensible limits each week, and a high frequency of at-risk consumption was observed. Another recent study conducted in Italy^(17)^ also shows a high percentage of high-risk (53·3 %) drinkers.

Furthermore, in our series, risk alcohol consumption is associated with smoking and with higher medical courses, which could point to poor stress management among our students. All this suggests the need to carry out an intervention program with the aim of reducing alcohol consumption and making future health professionals aware of the risks of alcohol and other toxic habits such as smoking. It is important to know about alcohol use in students, and to make them aware of its risks, especially in future health professionals.

Regarding physical activity, a highly significant association with MeDi was observed in our study. Several studies have shown an association between adherence to MeDi and physical activity in children and adolescents^(37)^. In addition, several studies support that good physical fitness and high adherence to MeDi are associated in isolation and in combination with a higher quality of life and lower morbidity and mortality^(50)^.

### Limitations and strengths

This study has several limitations. The cross-sectional nature of the study precludes establishing causality. The fact that the sample consisted of medical students and the voluntary nature of the survey may have influenced those included in the study were more concerned about a healthy diet. Furthermore, the use of questionnaires as a method of dietary assessment is limited by the fact that veracity of the data depends on the correct understanding of the questions and the accuracy of the information provided. Finally, no analytical determinations nor information on additional determinants of dietary choices such as economic or social determinants were collected. In relation to alcohol consumption, not having established the type of alcohol consumed (fermented or distilled beverage) limits the interpretation of the data.

Despite these limitations, the large sample size of the study and the non-exclusion of any student from participating allows us to obtain representative data on adherence to MeDi by medical students. Another strong point is the participation of students from all courses, which enables us to analyse the differences between them.

## Conclusions

MeDi compliance by medical students was 58·9 %, being significantly higher at older age and in higher academic years. It was also significantly related to greater physical activity.

This could support the greater knowledge about nutrition and diseases, the greater dietary compliance. However, it would be useful to quantify the dietary knowledge of medical students and see its direct relationship with a better dietary habit.

It should be noted that almost one in two medical students have a risky alcohol consumption and one-third did not engage in regular physical exercise. This added to the above suggests the need to implement early nutritional and healthy lifestyle educational programs for university students in general and even more so for future health professionals.
